# Clinical Utility of Soluble Lectin Type Oxidized Low-Density Lipoprotein Receptor as a Biomarker for Myocardial Infarction and Stable Angina

**DOI:** 10.7759/cureus.50719

**Published:** 2023-12-18

**Authors:** Radhakishan Narsini, Vijaya Bhaskar, Hajra Luqman, Sai Satish O, Shyam Sundar R Parupati, Ranga Reddy A B.V, Iyyapu Krishna Mohan

**Affiliations:** 1 Biochemistry, Nizam's Institute Of Medical Sciences, Hyderabad, IND; 2 Biochemistry, Nizam’s Institute of Medical Sciences, Hyderabad, IND; 3 Cardiology, Nizam’s Institute of Medical Sciences, Hyderabad, IND; 4 Cardiology, Krishna Institute of Medical Sciences (KIMS) Hospitals Kondapur, Kondapur, IND; 5 Cardiology, Apollo Hospitals, Hyderabad, IND; 6 Biochemistry, Nizam's Institute of Medical Sciences, Hyderabad, IND

**Keywords:** slox-1, soluble lectin-type oxidized low-density lipoprotein receptor 1, cardiac troponin, stable angina, plaque rupture, myocardial infarction, lectin type oxidized ldl receptor 1

## Abstract

Background and objectives

Endothelial soluble lectin-type oxidized low-density lipoprotein receptor 1 (sLOX-1) recognizes oxidized low-density lipoprotein (LDL) and triggers downstream signaling leading to atherosclerosis. The objective of this study was to demonstrate the utility of sLOX-1 as a biomarker for detecting acute myocardial infarction (MI) and stable angina (SA) and to develop a diagnostic algorithm for distinguishing coronary vasospasm from coronary plaque rupture.

Methods

We enrolled 62 patients who underwent diagnostic coronary angiography (CAG) and 30 healthy controls (21 men and nine women) and measured sLOX-1, troponin I, and cardiac myosin-binding protein C (c-MyBPC) using commercial kits.

Results

Patients with MI exhibited higher sLOX-1 levels (301.55 ± 196.16 pg/ml) than patients with stable angina (220.76 ± 103.65 pg/ml) and healthy controls (121.14 ± 77.10, F: 10.55, p<0.001). Although higher troponin I levels were detected in MI patients (263.00 ± 493.00 vs. 3.19 ± 2.15 ng/ml, p=0.0019), no significant elevation was observed in SA patients (1.91 ± 0.79 ng/ml). Plasma sLOX-1 levels showed a positive association with age (r=0.37, p=0.003), but not with gender (r=0.04, p=0.75). Troponin I showed no association with age (r=0.12, p=0.36) or gender (r=0.06, p=0.62). Receiver operating characteristic (ROC) curves revealed that among the three biomarkers, troponin-I showed a higher area under the curve (AUC) (AUC: 0.941), followed by sLOX-1 (AUC: 0.888), while c-MyBPC showed no clinical utility in the detection of MI (AUC: 0.666).

Conclusions

A marked elevation of sLOX-1 can detect MI and differentiate the presence or absence of plaque rupture, along with diagnosing stable angina.

## Introduction

Myocardial infarction (MI) is one of the most common cardiovascular disorders associated with a high degree of mortality. Its incidence is 3.8% and 9.5% in subjects with ages <60 years and >60 years, respectively [1]. High-sensitivity troponins like (hs)-troponin T and hs-troponin I, natriuretic peptides, mid-regional pro-adrenomedullin, galectin-3, and soluble suppression of tumorigenicity are currently recommended biomarkers for MI [2]. Among these, hs-troponin T and hs-troponin I are the most widely used markers for diagnosing MI in hospitals. These two markers together exhibit a pooled sensitivity of >90%. Troponin I had better specificity compared to troponin T in the early diagnosis of acute MI (80% vs. 68%) [3].

Lectin-type oxidized low-density lipoprotein receptor 1 (LOX-1) is a 50 kDa transmembrane protein present in endothelial cells that recognizes oxidized low-density lipoprotein (ox-LDL) and triggers downstream signaling leading to the acceleration of atherosclerosis via endothelial dysfunction, increased uptake of ox-LDL, and apoptosis [4]. Lectin-type oxidized low-density lipoprotein receptor 1 is expressed in platelets and cardiomyocytes, thus enhancing platelet activation, promoting their adhesion to endothelial cells, facilitating their aggregation, and inducing ischemic insult by developing cardiac fibrosis and myocyte apoptosis [4].

Patients with coronary slow flow have shown a significant elevation in serum soluble LOX-1 (sLOX-1) correlating with thrombolysis in MI frame count [5]. A significant elevation in plasma sLOX-1 levels was reported in non-ST segment elevated MI (NSTEMI) and ST segment elevated MI (STEMI) patients, with corresponding area under the curve (AUC) values of 0.92 and 0.925, respectively [6]. In a 62-year-old woman with mild STEMI and mildly increasing troponin T, sLOX-1 levels did not increase during hospitalization, thus helping in the diagnosis of the MI as coronary vasospasm and ruling out the possibility of coronary plaque rupture [7]. A follow-up for 1,156 days in STEMI patients revealed that elevated sLOX-1 levels increase the risk of all-cause mortality and major adverse cardiovascular events by 5.89 and 3.46-folds, respectively [8]. In NSTEMI and unstable angina, elevated high-sensitivity C-reactive protein (hs-CRP) and sLOX-1 levels are associated with the modified Gensini scores [9].

Compared to NSTEMI patients, STEMI patients were found to have higher circulating levels of sLOX-1 and a higher ratio of sLOX-1 to membrane-bound LOX-1 in aspirated thrombi, with a parallel increase in the most negative low-density lipoprotein 5 (L5) [10]. Circulating sLOX-1 levels are elevated in patients at risk of periprocedural myocardial infarction (PCI-RPMI) undergoing elective native single-vessel percutaneous coronary intervention [11].

Elevated sLOX-1 levels are shown to persist for 24 hours after admission, while other markers such as heart-type fatty acid-binding protein (H-FABP), myoglobin, troponin T, and creatine kinase-myoglobin binding (CK-MB) were not elevated at the time of admission and peaked only after 2 hours [12]. The diagnostic sensitivity of sLOX1 was 93%, while other markers showed sensitivity ranging from 33% to 78%. Plasma sLOX-1 levels were reported to be higher in cases with MI compared to unstable angina and controls with a sLOX-1/oxidized LDL ratio showing higher discriminatory ability [13].

Despite these advances and the documented clinical utility of sLOX-1 as one of the key biomarkers of atherosclerosis and MI, only one study has been reported from India. This study demonstrated the association of sLOX-1 with coronary artery disease (CAD), with a sensitivity of 87.88% and a specificity of 100% [14]. Post-interventional sLOX-1 levels served as indices of recurrent coronary artery disease or acute coronary syndrome [14]. The objective of the current study was to demonstrate the utility of sLOX-1 as a biomarker for detecting acute MI and develop a diagnostic algorithm for distinguishing coronary vasospasm from coronary plaque rupture using a combination of sLOX-1, cardiac myosin-binding protein C (c-MyBPC), and troponin I.

## Materials and methods

This study was a cross-sectional observational study. We enrolled 62 patients who underwent diagnostic coronary angiography (CAG) at the cardiology department of Nizam’s Institute of Medical Sciences, Hyderabad, India. Patients were recruited based on their availability during the study period. Myocardial infarction and stable angina (SA) were diagnosed in 50 (40 men and 10 women) and 12 patients (four men and eight women), respectively. To prove sLOX-1 as an early indicator /biomarker of SA, which is an initial event of CAD, parallelly, we enrolled 30 healthy controls (21 men and nine women). The study was conducted between January 2021 and December 2022.

Inclusion and exclusion criteria

All the patients who were newly diagnosed with MI and attended our hospital within six hours after the event were included in the study. Six hours were calculated based on the initiation of chest pain. Patients with unstable angina, symptomatic peripheral artery disease, cerebrovascular stroke, and patients who did not give informed consent were excluded from the study.

Informed consent was obtained from all these patients. The mean ages at diagnosis of MI and unstable angina were 54.9 ± 11.7 years and 43.8 ± 18.1 years, respectively. Thirty-three subjects had MI at <60 years of age, while 17 of them had MI at >60 years. This study, carried out following the principles of the Declaration of Helsinki, was approved by the Institutional Ethical Committee of Nizam’s Institute of Medical Sciences, Hyderabad, India (ESGS No. 797/2019).

Sample collection and testing

The blood samples were collected in a plain vacutainer during coronary angiography for patients undergoing CAG or at the time of enrollment for the healthy controls. The serum was separated within half an hour and stored at -80°C until sLOX-1 and c-MyBPC assays were performed.

Measurement of sLOX-1 and other biomarkers

A commercially available quantitative sandwich enzyme-linked immunosorbent assay (ELISA) kit was used to determine serum sLOX-1 levels as per the manufacturer’s instructions. The inter-assay and intra-assay precisions were 8.9% and 7.8%, respectively. Levels of c-MyBPC and troponin I were determined on the same serum samples as those for sLOX-1 by commercially available ELISA kits (Krishgen Biosystems, Cerritos, CA).

Statistical analysis

A Student’s t-test was used to establish the association of continuous variables with MI based on the difference in the distribution of these variables between cases and controls. Analysis of variance (ANOVA) was used to compare the variables across three groups, i.e., MI, SA, and controls. Receiver operating characteristic (ROC) curves were plotted with 1-specificity on the X-axis and sensitivity on the Y-axis. Area under the curve statistics were used to assess the diagnostic utility. Pearson’s correlation was used to establish the correlation of age, gender, and biomarkers with each other.

## Results

**Figure 1:**
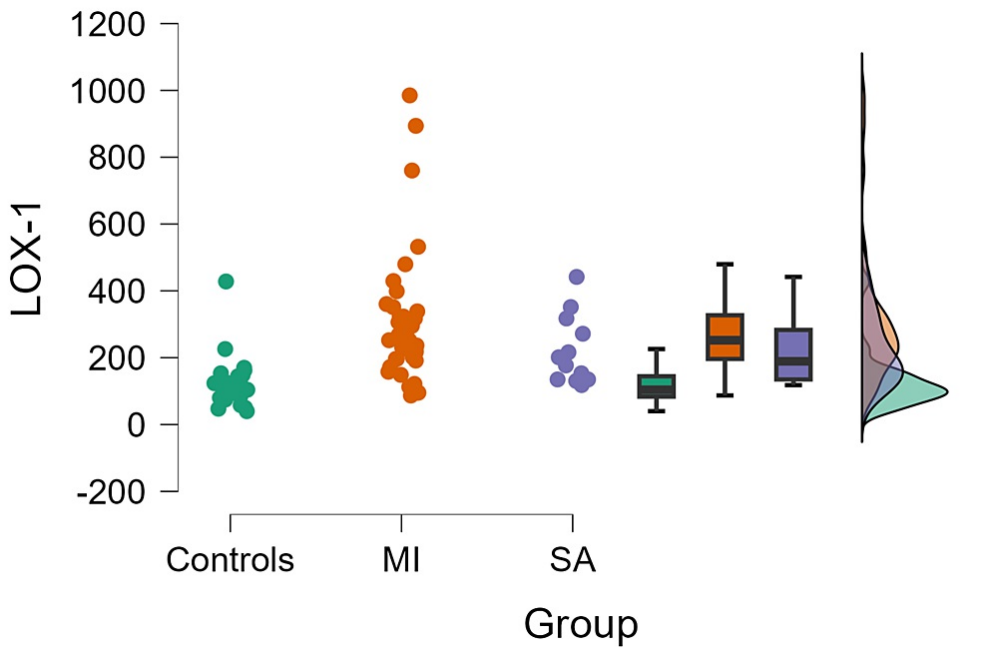
Association of sLOX-1 with myocardial infarction LOX-1: lectin-type oxidized low-density lipoprotein receptor-1: MI: myocardial infarction; SA: stable angina

The sLOX-1 and troponin I levels were measured and compared between cases and controls. Patients with MI exhibited higher sLOX-1 levels (301.55±196.16 pg/ml) than patients with SA (220.76±103.65 pg/ml) and healthy controls (121.14±77.10, F: 10.55, p<0.001) (Figure 1).

**Figure 2:**
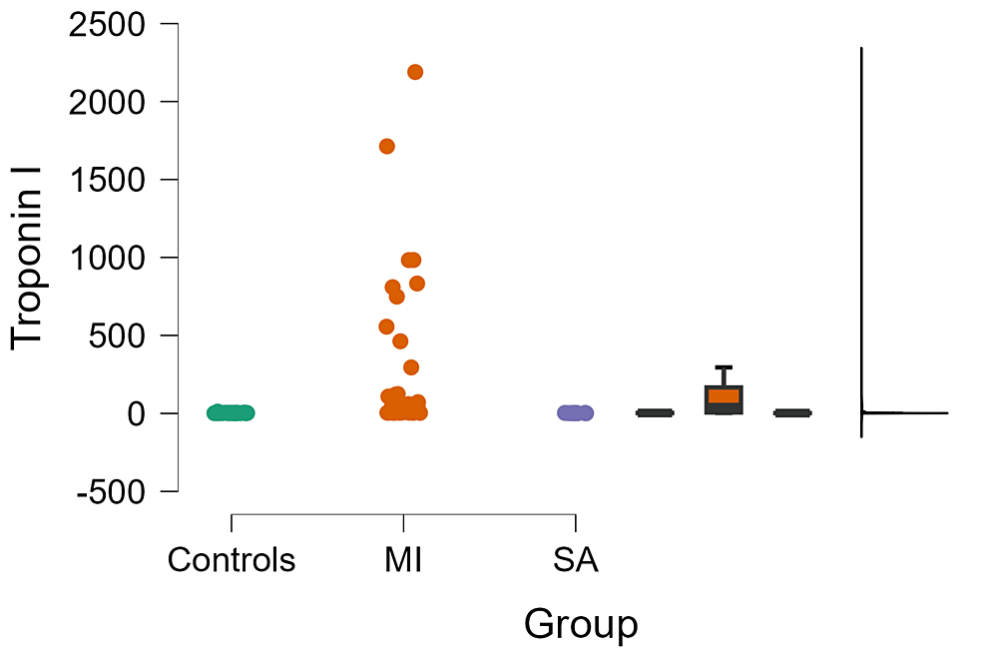
Association of troponin-I with myocardial infarction MI: myocardial infarction; SA: stable angina

Patients with MI exhibited higher troponin I levels when compared to controls (263.00±493.00 vs. 3.19±2.15 ng/ml, p=0.0019). However, no elevation of troponin I was observed in SA patients (1.91±0.79 ng/ml) (Figure 2).

**Figure 3:**
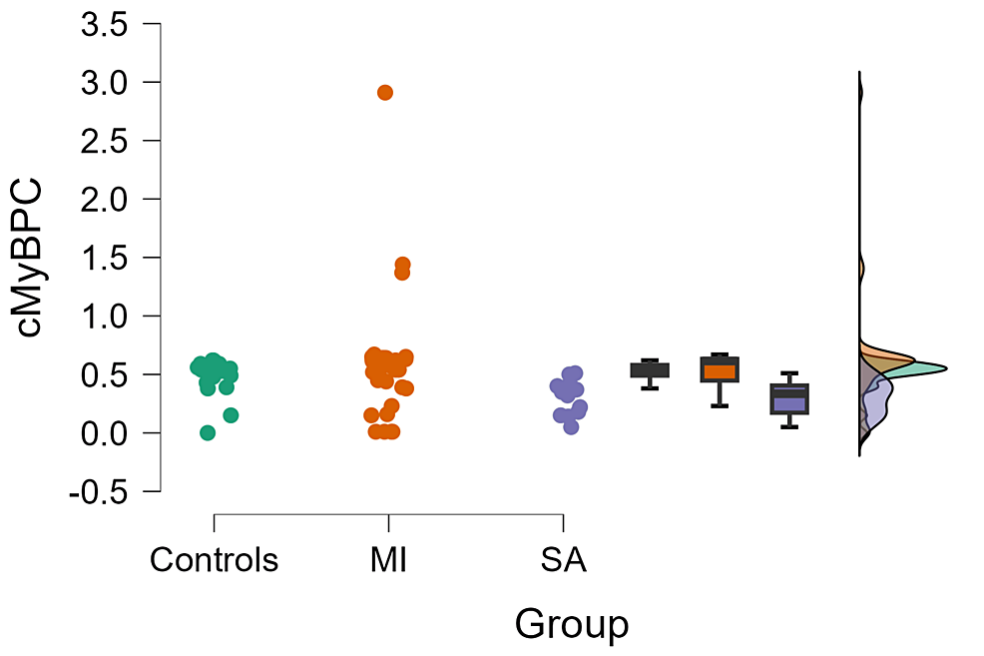
Association of c-MyBPC with myocardial infarction c-MyBPC: cardiac myosin-binding protein C; MI: myocardial infarction' SA: stable angina

Also, C-MyBPC levels were measured and compared between cases and controls. No statistically significant differences were observed in c-MyBPC levels among MI (0.60±0.47) and SA cases (0.30±0.15) and controls (0.50±0.14, p=0.22) (Figure 3).

Correlation of biomarkers with demographic characteristics

Plasma sLOX-1 levels showed a positive association with age (r=0.37, p=0.003), but not with gender (r=0.04, p=0.75). Linear regression revealed a 22.32 pg/ml elevation in sLOX-1 levels per decade of life in MI patients, while this increase is only 6.617 pg/ml per decade in healthy controls. Troponin I showed no association with age (r=0.12, p=0.36) or gender (r=0.06, p=0.62). The c-MyBPC levels were lower in men compared to women (p=0.025), but not associated with age (r=0.01, p=0.93).

Diagnostic utility of biomarkers

**Figure 4:**
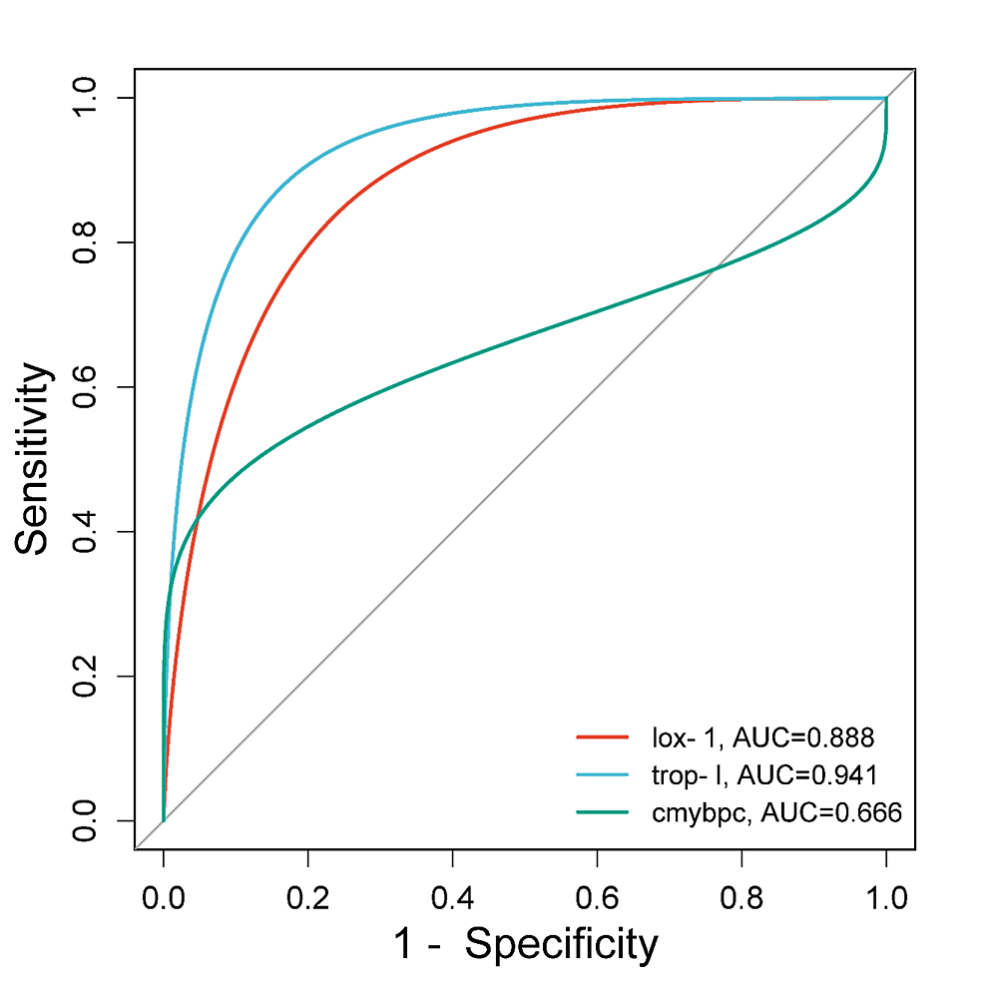
Receiver operating characteristic curves to demonstrate the clinical utility of biomarkers lox-1: lectin-type oxidized low-density lipoprotein receptor 1; trop-I: troponin-I; c-MyBPC: cardiac myosin-binding protein C; AUC: area under the curve

The ROC curves revealed that among the three biomarkers, troponin-I showed higher AUC levels (AUC: 0.941), followed by sLOX-1 (AUC: 0.888), while c-MyBPC showed no clinical utility in the detection of MI (AUC: 0.666) (Figure 4).

**Figure 5:**
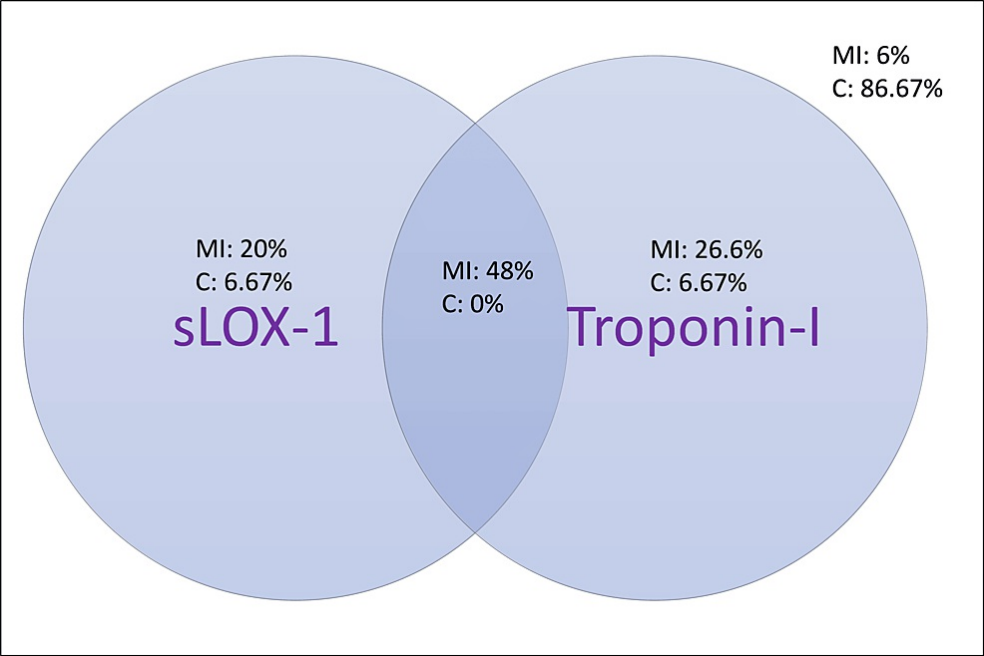
Clinical utility of sLOX-1 and troponin I in diagnosing myocardial infarction sLOX-1: lectin-type oxidized low-density lipoprotein receptor-1; MI: myocardial infarction; C: controls

Using the 95th percentile of sLOX-1 and troponin I in the controls as the cut-off, we plotted the Venn diagram to understand the clinical utility of these markers in different combinations. Both biomarkers were below the cut-off in 6% of MI cases and 86.67% of controls. Only sLOX-1 elevation was observed in 20% of MI cases and 6.67% of controls (odds ratio (OR): 43.33, 95% CI: 6.28-299.18, p<0.0001). Only troponin I elevation was observed in 26.6% of MI cases and 6.67% of controls (OR: 56.33, 95% CI: 8.3-380.06, p<0.0001). Both markers are elevated in 48% of MI cases and none of the controls. The positive predictive value is 100%, and the negative predictive value is 89.7% if both markers are elevated above the cut-off (Figure 5).

## Discussion

In the current study, we have demonstrated that sLOX-1 levels serve as biomarkers for MI in addition to troponin I. The levels of c-MyBPC showed a null association with myocardial infarction. In stable angina, sLOX-1 levels are elevated. With the increase in each decade of age, sLOX-1 levels were shown to increase. AUC statistics revealed the good clinical utility of sLOX-1 in addition to troponin I in the diagnosis of myocardial infarction. We also demonstrate that the positive predictive value of MI increases to an absolute 100% if both sLOX-1 and troponin I are measured. In cases with stable angina, sLOX-1 levels are higher than controls, but troponin I levels are not elevated, thus justifying its utility in distinguishing stable angina based on both biochemical markers.

Systolic blood pressure, increasing age, heart rate, and sLOX-1 were reported as independent determinants of arterial stiffness [15]. In a Japanese study, sLOX-1 was shown to predict acute coronary syndrome more accurately (AUC: 0.948) than troponin T (AUC: 0.704) and H-FABP (AUC: 0.691) [16]. No gender differences were observed in the distribution of sLOX-1 levels in this study, corroborating our findings. The well-documented risk factors such as diabetes, smoking, and hypertension also showed no significant association with sLOX-1 [16].

Markstad et al. demonstrated that ox-LDL induces the release of sLOX-1 from endothelial cells, and hence, sLOX-1 circulating levels correlate with carotid plaque formation and the risk of ischemic stroke [17]. Caloric restriction and moderate-to-vigorous intensity exercise for 20 weeks were shown to be effective in reducing the sLOX-1 levels by 23%, with subsequent reductions in weight, body fat, and waist and hip ratio [18]. A drop in sLOX-1 levels served as a good prognostic indicator. In acute coronary syndrome patients undergoing intracoronary imaging and statin therapy, elevated levels increased the risk of mortality by 3.81-fold [19]. Compared to troponin T and hs-CRP, sLOX-1 levels were reported to distinguish acute coronary syndrome cases with plaque rupture from those without plaque rupture. Cases with plaque rupture show a marked elevation of sLOX-1 [20].

The major strengths of the current study are the parallel measurements of troponin I, sLOX-1, and c-MyBPC in the patients with MI, which helped in comparing the utility of sLOX-1 along with troponin I. Large studies are warranted to substantiate these findings further. The inclusion of sLOX-1 as a biomarker helps not only in the diagnosis but also in distinguishing the presence or absence of plaque rupture, serving as a prognostic indicator that helps prevent the recurrence of adverse cardiovascular events and death.

Limitations of the study

The limitations of the present study are that the sample size was small. Therefore, the results may not be representative of the population at large. Also, we had not included the unstable angina cases.

## Conclusions

To conclude, sLOX-1 is markedly elevated in MI and has good clinical utility in diagnosing and differentiating coronary vasospasm from plaque rupture. Elevation of sLOX-1 without elevation of troponin I will help identify cases with stable angina. Regular monitoring of sLOX-1 levels may be necessary as a prognostic indicator and as an index of response to therapy. Troponin I and sLOX-1 together increase the positive predictive value for MI.
